# Cerebral organoids: emerging ex vivo humanoid models of glioblastoma

**DOI:** 10.1186/s40478-020-01077-3

**Published:** 2020-12-01

**Authors:** Michail-Dimitrios Papaioannou, Kevin Sangster, Rifat Shahriar Sajid, Ugljesa Djuric, Phedias Diamandis

**Affiliations:** 1 Princess Margaret Cancer Centre, 101 College Street, Toronto, ON M5G 1L7 Canada; 2 Laboratory Medicine Program, Department of Pathology, University Health Network, 200 Elizabeth Street, Toronto, ON M5G 2C4 Canada; 3 Department of Laboratory Medicine and Pathobiology, University of Toronto, Toronto, ON M5S 1A8 Canada; 4 Department of Medical Biophysics, University of Toronto, Toronto, ON M5G 1L7 Canada

**Keywords:** Glioblastoma, Cerebral organoids, Cancer modelling, Cancer discovery

## Abstract

Glioblastoma is an aggressive form of brain cancer that has seen only marginal improvements in its bleak survival outlook of 12–15 months over the last forty years. There is therefore an urgent need for the development of advanced drug screening platforms and systems that can better recapitulate glioblastoma’s infiltrative biology, a process largely responsible for its relentless propensity for recurrence and progression. Recent advances in stem cell biology have allowed the generation of artificial tridimensional brain-like tissue termed cerebral organoids. In addition to their potential to model brain development, these reagents are providing much needed synthetic humanoid scaffolds to model glioblastoma’s infiltrative capacity in a faithful and scalable manner. Here, we highlight and review the early breakthroughs in this growing field and discuss its potential future role for glioblastoma research.

## Introduction

### A disease with a dismal prognosis

Glioblastoma multiforme (GBM) is a malignant primary brain tumor with a dismal 5-year survival rate of 5% and median survival of just 14 months [1]. Developments of effective treatments are challenged by GBM’s extensive cellular heterogeneity and its ability to infiltrate into surrounding brain tissue, making complete surgical resection infeasible [2]. The majority of GBM tumors recur, partly hypothesized to be due to the presence of a chemo-resistant stem-cell-like population that repopulates the tumor following therapy [3, 4]. Despite numerous drugs reaching clinical trials, the standard of care for GBM treatment has remained largely unchanged for almost two decades [5]. As such, there has been significant interest in refining and expanding laboratory models of GBM in an effort to better recapitulate its true biology, and in turn, help identify new and more effective therapies. Emerging models that combine synthetic tridimensional human neural cultures, known as cerebral organoids, with genetically tagged glioma stem cells have provided new tools for scientists to recapitulate the cellular and molecular heterogeneity of GBM. This includes both its ability to interact and infiltrate within normal brain tissue structures providing an exciting emerging model to study this deadly disease. Here, we compare and contrast this model with traditional systems and explore its complementary value in GBM research and discovery.

### Traditional models of GBM

Primary GBM cell cultures have been crucial in understanding the biology of this disease by providing a widespread and accessible in vitro model. Following the successful isolation and characterization of glioblastoma stem cells (GSCs), that promote cancer progression and recurrence [6, 7], this in vitro system has offered significant insight into fundamental mechanisms of cellular drivers of GBM. However, these culture systems have come with limitations and compromise as they were not designed to capture complex three-dimensional aspects of GBM’s anatomical architecture, their interactions with native non-neoplastic tissue structures or its characteristic infiltrative behavior [8]. Recent research has also highlighted discrepancies observed when these systems are leveraged for drug discovery, with discordances in the efficacies of drugs in experimental and clinical settings [9]. Also absent from these systems are the reciprocal interactions between the tumor and healthy neural tissue. These interactions, such as the recently demonstrated electrical and synaptic integration of gliomas into surrounding neural tissue, have been shown to be especially relevant to the growth and progression of GBM [10–12]. These studies further highlight the need for models that incorporate the well-established role of the tumor microenvironment to allow research into escape mechanisms of GBM through invasion and host interactions.

Animal models have provided some solutions to the limitations of these in vitro cultures by providing a more holistic system for dissecting GBM molecular and cellular biology by incorporating tumor-host interactions [13]. Of these models, genetically engineered mouse models have been particularly useful in understanding GBM but are limited by substantial differences between human and mouse brains [14]. Furthermore, results from these models unfortunately have a history of being imperfect predictors of treatment outcomes in human clinical trials [15–17]. Moreover, recent studies using patient-derived xenograft (PDX) models showing a tendency for human-derived GSCs to undergo murine-specific changes, that diverge from human tumor biology, have also challenged these systems and potentially explain challenges in translating discoveries from that model to the clinic [18]. Along with genetically engineered mouse models, these PDX models also suffer from high costs and an incompatibility with medium to high-throughput drug screens. These limitations highlight the need for complementary tools that can serve as scalable and faithful models of human GBM biology including an ability to potentially capture the critical tumor-host interactions central to this disease.

## Main Body

### Early ex-vivo models of GBM and interactions with other cell types

Initial efforts to improve ex vivo modelling of GBM focused on examining the role of extracellular matrix components in cancer growth dynamics [19] and were successful in increasing the longevity and size of three dimensional GSC populations by embedding them in Matrigel [20]. These larger cell aggregates could be cultured for months and generated hypoxic niches that were enriched in stem like-cells that were radioresistant [20]. More recent efforts have also optimized a fast and efficient protocol for generating tumor “organoids” out of small primary tumor pieces that can be grown and stored in catalogued biobanks, whilst preserving the genetic profile and cellular heterogeneity of the original tumor [21]. Together these provide more complex models to study intra-tumoral heterogeneity of GBM in human exclusive cell systems. Alternatively, the development of scalable models that incorporate non-neoplastic cell-based compartments was addressed initially by efforts that co-cultured engineered neural tissue from embryonic stem cells with GSC spheres [22]. Even though early neuronal tissue did not provide the cellular heterogeneity present in the adult brain, this study was important as being the first to show that GSCs had the ability to interact with other human cell types in vitro. The issue of cellular heterogeneity was addressed in a recent study [23] that optimized a three-dimensional brain microphysiological cell protocol that could generate mature neurons and glial cells [24]. Plummer and colleagues added GSCs on a layer of neural precursor cells with the resulting hybrid spheres containing growing tumor cells, but critically did not show the characteristic invasive pattern of clinical GBM [23]. It is possible that the perturbed developmental programming of neural precursors, due to the addition of GSCs, resulted in a microenvironment that did not mimic the one present in the adult brain.

### Cerebral organoids as an emerging model system

Cerebral Organoids (COs) are one of the most promising recent developments which attempt to provide an accurate microenvironment into which GSCs can infiltrate. These represent complex 3D cell aggregates derived from human pluripotent stem cells (hPSCs) which undergo neural differentiation and self-organize to form layered structures that resemble the developing human brain. COs have recently emerged as a model system for studying CNS disease within a human genetic background, as they appear to better recapitulate the cell diversity of the human cerebral cortex, including populations of neural progenitor cells, astrocyte precursor cells, oligodendrocyte precursor cells, excitatory neurons, inhibitory neurons and retinal cells [25, 26]. With remarkable structural similarities to the human brain, COs can also exhibit characteristics of cortical tissue architecture such as cell-type specific layering [27], and even cortical folding [28]. Recent efforts have also generated choroid-plexus-like organoids with a selective barrier resembling that of the blood–brain barrier. In addition to the ability to model production of cerebrospinal fluid, these organoids potentially allow for scalable assessment of a candidate drugs’ ability to cross the blood–brain barrier [29]. COs have been used to model brain development as well as a variety of diseases including microcephaly, lissencephaly, autism spectrum disorder, and even neurodegenerative disorders such as Alzheimer’s [30].

**Fig. 1:**
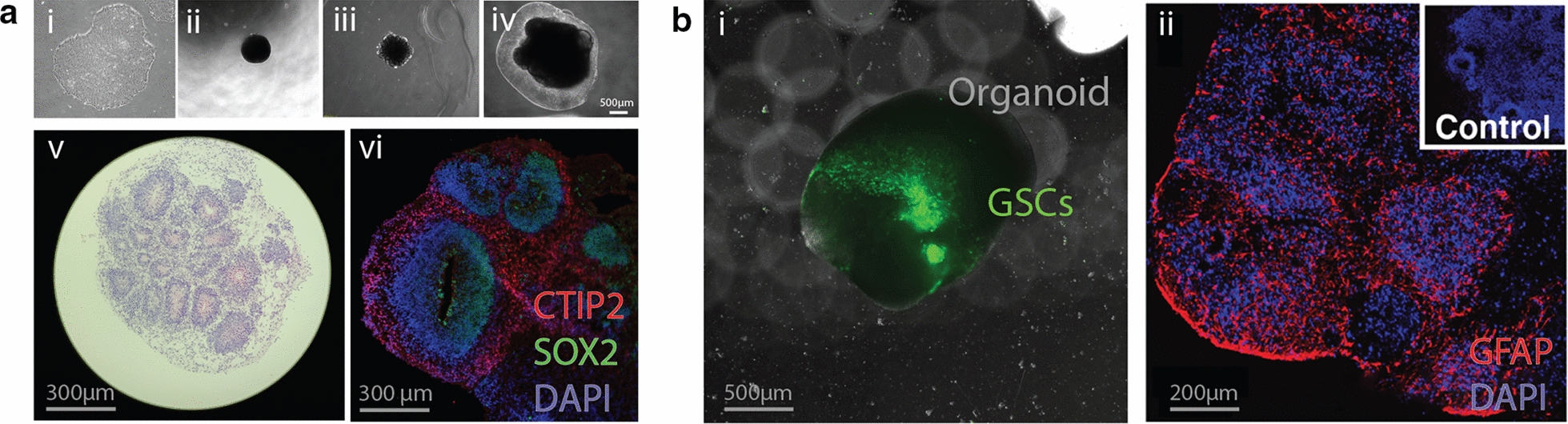
Illustative example of how cerebral organoids are generated and their use for modeling GBM infiltration. **a** Typical developmental outline of cerebral organoid formation across time (i–iv) and validation with neuronal markers using histology (v) and immunofluorescence (vi) at 6 weeks to highlight formation of spatially organized brain-like tissue. Markers shown in immunofluorescence include SOX2 (primitive neuroepithelial progenitor cells), DCX (early neurons) and DAPI as a nuclear stain. **b** Characteristic epifluorescence image (i) of a fused GBM-organoid culture system through co-culturing of cerebral organoids with GFP-tagged GSCs. In (ii) immunofluorescence indicates the level of infiltration of GBM into neuronal tissue. In (ii), GFAP is used as a surrogate marker of the infiltrating GBM cells as it is not typically expressed in high levels in cerebral organoids at this timepoint (non-fused organoid in indent)

With the emergence of research pointing to GSCs being successfully used in more complex, fusion based culture protocols, recent studies have used human cerebral organoids as a model system to study GBM growth in a tridimensional human brain-like environment. These efforts can be broadly divided into two categories based on the method of inducing tumor formation in organoids. One is the use of genetically engineered organoid models, which are well suited for understanding genetic mechanisms of GBM induction within the 3D environment of healthy tissue that is present during in vivo tumorigenesis [31, 32]. The second relies on the introduction of patient derived GSCs into organoids, which is particularly well suited for understanding clinical characteristics following initial tumor formation. Due to the central role of GSCs as the cell type that drives tumor progression and recurrence [33], these efforts have focused on introducing GSCs into COs, either as single cells [34–37] or as GSC spheres [38]. Characteristic results of the latter method, which is utilized in our laboratory, are presented in Fig. 1.

### Co-culturing systems of GBM and cerebral organoids

After initial indications of the possibility for organoids being receptive to fusion [39], da Silva and colleagues introduced GSCs into cerebral organoids to model GBM [38]. They observed GSC spheroids consistently fusing, becoming incorporated within and exhibiting more infiltration in organoids when compared to fusions with neural progenitor spheroids. Additionally, immunofluorescence staining validated infiltration markers of GBM in this ex vivo model [38], despite using human-derived GSC spheroids and mouse-derived cerebral organoids. Given the differences in human and mouse brain structures [40], this co-culture system was not further used aside from being a proof-of-principle for future improvements. Subsequently, several other groups utilized the fusion approach as an ex vivo model of GBM [32, 34–37] and drug screening [31, 36].

A more recent study used stable lines of GFP tagged GSCs and co-cultured them with cerebral organoids to generate what authors called the “glioma cerebral organoid (GLICO)” model [36]. This system was observed to recapitulate features of human GBM behavior, namely the infiltration into normal brain tissue and the presence of 3D interconnecting tumor microtubes that were previously reported to serve as a key mechanism of tumor cell communication and resistance [41]. GLICOs were also able to maintain certain molecular signaling networks found in the actual tumors derived from patients, such as amplified epidermal growth factor receptor (EGFR) expression. Importantly, Linkous and colleagues observed differential sensitivity to chemotherapeutic agents in 2D GSC cell lines compared to GLICOs [36]. The latter presented a response more closely related to clinical observations, thereby making a case for the CO model’s ability to more closely recapitulate the true biology of GBM. Interestingly, this successful tumor cell invasion in the fusion model identified differentially expressed genes during this process [35]. Quantification of invasion from different GSC lines supported the capability of the fusion model to reproduce the clinical invasiveness properties of the original parent tumors. To next examine changes in gene expression of tumor cells during invasion, GSCs were co-cultured with cells from dissociated organoids and analyzed using single-cell RNA sequencing. These studies showed an upregulation of model-validating processes with invasion-related genes, as well as ligand-receptor pairs between interacting tumor and normal cells. These experiments, and the datasets generated, may also offer new insights, key molecular participants and targets for tumor invasion. However, it is important to note that these experiments didn’t faithfully recapitulate GBM behavior since the interaction between tumor and normal cell populations was brief and not in the context of brain tissue infiltration.

A similar ex vivo approach was used in another study that explored the diverse cell type composition of GBM tumors [34]. This study focused on a neuro-developmental cell type called outer radial glia that are believed to be reactivated in GBM and contribute to its heterogeneity. Similar to the developmental cell type, the outer radial glia-like tumor cells also express the cell surface marker PTPRZ1. To better understand the tumorigenic properties of these cells in the context of a human tissue microenvironment, Bhaduri and colleagues transplanted PTPRZ1 +/GFP + cells from primary tumors into cerebral organoids [34]. This led to invasion and expansion of the recipient organoids. The tumor cell population that was isolated from the fused culture was found to be composed primarily of neurons and astrocytes, which resembles the heterogenous composition of the initial primary tumor. The results of this study emphasize the strength of the fusion model in recapitulating the nature of GBM, specifically in terms of tumor cell type composition. This could be potentially due to successful mimicking of the tumor microenvironment forming between the fused cell populations.

Lastly, a recently published study compared different GBM models and their ability to reproduce cellular states found in primary tumors by single-cell RNA sequencing profiles sourced from four models: glioma spheres, tumor organoids, orthotopic PDXs, and GLICOs [37]. These results indicated that the composition of primary tumor cellular states was most similar to the one found in GLICOs. Moreover, the single cell signatures of GLICOs contained a greater diversity of cell types that were also found in primary tumors. Further analysis of their transcriptomic data showed that expression of Notch pathway members and GBM invasion genes was upregulated in a greater number of cells in GLICOs compared to other models, and the expression of certain Notch ligands was also increased in non-tumor cells of the GLICO. The dependency of these profiles on the presence of the organoid compartment in the model was further supported when GSCs derived from GLICOs were isolated and grown in 2D culture. Indeed, single-cell RNA sequencing of these cells revealed that they more closely resembled glioma spheres than primary tumors. These results not only underscore the critical interaction between tumor and normal cells for reproducing cellular states in GBM modelling, but also highlight the need for a more neuroanatomically accurate human microenvironment, which currently can only be achieved through the leveraging of cerebral organoids.

### Genetically engineered organoid models of GBM

As an alternative to using patient-derived tumor cells, some groups have opted to model GBM in cerebral organoids by using genetic manipulations to induce tumorigenesis [31, 32]. This mirrors the commonly used approach of modeling GBM in genetically engineered mouse models and is well suited for studying early genetic events leading to tumor formation of human gliomas. One such study utilized CRISPR/Cas9 to simultaneously disrupt the tumor suppressor TP53 and insert the oncogene HRas^G12V^ by homologous recombination [32]. Although the HRas^G12V^ mutation itself is not a common feature of GBM, through this manipulation the authors were able to experimentally simulate Ras pathway overactivation, a common molecular feature of GBM biology [42]. As a proof of principle, this study demonstrated the ability to generate bona fide tumors that were highly invasive and proliferative. Transcriptomic profiles of the organoid-derived tumor cells were compared to established GBM subtypes and were found to resemble the mesenchymal subtype [43], further adding validity to the capability of this system to mimic clinically aggressive GBM subtypes. The study [32] also demonstrated that these organoid-derived tumor cells were serially transplantable between organoids and were lethal when introduced to immunocompromised mice, where they exhibited the morphological and angiogenic qualities of tumors observed in vivo. This study served as an important proof-of-concept report for the generation of genetically engineered organoid models of GBM. While the authors only carried out one genetic manipulation, this technique has the potential to be applied to the full range of mutations and molecular alterations clinically observed in GBM.

Other examples of this approach include efforts by Bian et al. which combined transposon-mediated insertion of oncogenes with CRISPR-Cas9-mediated mutagenesis of tumor suppressor genes [31]. Using this approach, 18 single gene mutations/amplifications and 15 common mutation/amplification combinations were generated. From these, the authors identified 4 that were capable of generating highly invasive tumors that overgrew into the surrounding organoid. In addition to high expression of invasion-related genes in the formed tumors, the authors identified transcriptional changes reflective of epithelial-to-mesenchymal transition. They also demonstrated that tumor growth could be attenuated by targeted drug treatment in some of the mutation/amplification combinations. Finally, they showcased the suitability of their system for use in large-scale drug screening by modifying the tumor cells to express firefly luciferase. The resulting luciferase activity could then be used to easily monitor tumor size when comparing the efficiency of various compounds on altering GBM growth dynamics.

## Discussion

### Challenges and future directions

While organoid models have already begun addressing many of the long-standing limitations of existing GBM culture systems, they are not without challenges and limitations. High inter-organoid variability in both morphology and tissue identity remains a significant issue for CO-based disease models [27, 30]. Ongoing improvements in this area include growing organoids on microfilament scaffolds to maximize surface area that is thought to promote more uniform neuroectoderm formation and suppress unwanted mesoderm elements [27]. Further refinements include utilizing engineered extracellular matrix (ECM)-like materials to improve consistency [44]. These engineered materials have the potential to greatly improve inter-batch variability compared to currently used methods, which typically utilize Matrigel as an ECM scaffold [44]. Other strategies that are gaining support to promote more consistent and high throughput organoid generation include the use of microwells for uniform embryoid body formation [45] and more well-defined differentiation protocols that can reproducibly generate organoids containing brain-region-specific cell types [26, 46, 47]. Others have engineered miniaturized spinning bioreactors that allow for better availability of differentiation cues to the 3D culture and, as a result, more advanced maturation of neuronal subtypes [26]. Increasing consistency of cerebral organoids and their ability to mimic different brain regions is expected to improve the accuracy and reproducibility of CO-based models of GBM. As these protocols evolve and stabilize to their most reliable and robust versions, acquiring a consensus and widespread use across the scientific community, they will likely also provide further and refined insights about the molecular machinery responsible for GBM’s relentless invasiveness and growth potential. In addition, CO-based models of GBM have lacked development of vasculature, which plays an important role in GBM progression by providing additional migratory tracks for GBM cells to use in infiltrating into surrounding tissue [48]. The perivascular niche also plays an important role in GSC maintenance [49]. Fortunately, significant efforts are already being directed to alleviate this limitation. Some groups have had success inducing formation of primitive blood vessel-like structures through vascular endothelial growth factor (VEGF) treatment during organoid formation [50]. Other successful attempts utilized organoids formed from human embryonic stem cells (hESCs) engineered to ectopically express ETV2 [51], or a co-culture of organoids and endothelial cells [52]. The potential of these structures to model important interactions between GBM and the perivascular niche represent exciting opportunities to expand the scope of this emerging model system down a number of increasingly more complex avenues. However, as vascularized organoids have yet to be used in GBM modeling, the ability of these structures to successfully model such interactions has yet to be seen.

Another important limitation, when compared to in vivo counterparts, is the lack of microglia or other critical immune cells in organoid-based models of GBM. The presence of microglia has been shown in cerebral organoids generated through certain protocols and can likely be further optimized to increase enrichment for these cells [53]. However, this approach relies on the generation of mesoderm progenitors within the cerebral organoid, which may give rise to other mesoderm-derived, non-neuronal tissues and compromise existing advantages. Additionally, in its current state, the quantity of microglia in organoids generated by this method, varies significantly which could further exacerbate the existing issue of inter-organoid heterogeneity. Whether this approach can be successfully integrated into organoid models of GBM while maintaining sufficient inter-organoid consistency remains an open question. Recent complementary efforts have attempted to bypass this issue by separately differentiating microglia-like cells and subsequently co-culturing them with isogenic, brain-region specific organoids. Initial results from these approaches, have been encouraging and appear to support that such exogenous production of microglia does not compromise functional interactions between them and other neuronal cell types within the organoids [54]. Analogous approaches could take advantage of well-established protocols for induced pluripotent stem cell (iPSC)-derived microglia in efforts to recapitulate the immune microenvironment [55, 56]. Similarly to the case of microglia, other reports also describe protocols for the generation of additional immune cell types from stem cells, such as monocytes and macrophages [57], T lymphocytes [58] and granulocytes [59]. Whether these immune components can be consistently and successfully incorporated into cerebral organoids without affecting established neuronal activities and interactions within the organoids remains to be investigated. If such incorporation efforts are successful, the prospects of introducing these additional immune components to the tumor immune microenvironment in the hybrid GBM-cerebral organoid model could further fuel interest in this advanced hybrid system. These are exciting areas of future developments, as they would foster development of scalable models for the pre-clinical and robust testing of emerging novel or immunotherapy treatments. A recent study reported the treatment of GBM-derived organoids with EGFRvIII-specific CAR-T cells that successfully induced cell death, EGFRvIII antigen loss and T cell activation only in those organoids deriving from GBM samples with high expression of EGFRvIII, a commonly mutated variant in glioblastoma [21]. Even though these three-dimensional structures were wholly derived from GBM, the successful and functional interaction between them and the CAR-T cell treatment points towards an increased likelihood that the hybrid system will also be successfully utilized in similar investigations.

As discussed, there is a strong potential for the use of these hybrid organoid models in many areas ranging from basic research to clinical investigations. Of therapeutic interest, there are important prospects in the use of hybrid cerebral organoid/GBM systems as a platform for drug screening, including for targeting tumor invasion and therapy-resistant populations of glioma cells. Initial proof-of-principle experiments where these models were treated with well-established chemotherapy drugs [31, 36] show promise in their utility to assess drug efficacies in controlling invasion and growth of GBM within them. Therapy-resistant populations of GBM have also been tested with temozolomide in GBM-derived organoids [60]. Whether these GBM subpopulations will be successfully integrated in the hybrid model remains to be seen. Besides the aforementioned efforts, organoid systems have been significantly leveraged in drug screening studies whether for the purpose of elucidating mechanisms of synergistic toxicity in medulloblastoma models [61] or in the context of neurodegeneration [62, 63] and epilepsy [64, 65]. Adding more layers of cellular complexity is expected to improve accuracy and efficient modelling of disease. Another exciting possibility is utilizing patient-derived tumor samples and iPSCs derived from the same individual to generate patient-specific tumor avatars for personalized drug testing. There is also the potential for patient iPSC collection to occur concurrently with existing tumor organoid biobanking efforts [21]. This would enable researchers to generate large repositories of tumor and patient-matched cerebral organoids that could be used in comparisons between tumor and healthy tissue as well as tumor invasion studies. Such efforts would also enable concurrent testing of both drug efficacy and toxicity in genetically matched samples.

Up to now, published studies have had outputs focused on the genomic or transcriptional level of these new hybrid cell systems and have been successful in showing how they are distinctly different from earlier in vitro cultures of GBM. Importantly, studies have also shown that the profiles of GBM cells grown within such a system are closer to the molecular profiles of GBM cells isolated from primary tissue than to earlier culture protocols. Therefore, extending the outputs of this new model to other-omics technologies, such as proteomics and epigenomics, seems the natural step that could combine with functional studies to provide concrete candidates for therapeutics. Medium throughput chemical or CRISPR-based screens can also be leveraged towards the same goal. To this end, further optimizations such as the integration of tissue clearing and quantitative 3D imaging will likely continue to increase the potential throughput of this system [66]. Whether any novel candidates or insights that might come out from these efforts will prove effective in slowing the progression of the disease in the clinic remains to be seen. If successful, this approach can potentially be expanded to other cancers with poor outcomes in need of an alternative path to generate new and meaningful therapies. Table 1 below summarizes the different approaches for the generation of this novel ex vivo model along with their strengths and weaknesses and corresponding references.

**Table 1:** Summary of the different ex vivo models of GBM

Model type	Pros	Cons	References
	Better captures clinical characteristics	Inconsistency during fusion process	[32, 34–38]
	More genetically diverse	Preparing of GSCs outside of tumor microenvironment	
	Two distinct populations that can be isolated/studied	Limited availability of primary cell lines	
	More amenable to optimization	Not applicable for studying tumor initiation events	
	Suitable for the identification of early genetic events leading to tumor formation	More genetically defined	[31, 32]
	Better discovery tool/uncovering mechanisms	Possible off-target effects need to be assessed	
	Allows for targeted studies of well-known GBM mutations	Plasmid transduction efficiency issues	
	Can address all possible trajectories of GBM formation	Requires validation of tumor presence	
	More focused study of extracellular matrix role	Does not mimic the human tumor microenvironment	[19–24]
	Faster turnaround of results	No three-dimensional architecture	
	Can incorporate novel elements of cell engineering	Interaction between tumorous and non-tumorous cell components not confirmed	
	Easier to add additional cell types	Harder to accomplish fusion/infiltration	

## Conclusion

The lack of significant improvement in GBM outcomes over recent decades spanning numerous breakthroughs in our understanding of the cellular and molecular mechanisms of cancer supports the continued search for alternative approaches to model and study this aggressive disease in the laboratory. Here we review the evolution and use of well-established stem cell protocols to generate tridimensional neuronal tissue with a cellular diversity similar to the human brain, particularly in the form of cerebral organoids. These dynamic cell aggregates are highly amenable to a wide array of experimental manipulations, including their ability to fuse with GSCs or engender a GBM-like cellular phenotype through genetic engineering. Initial studies have validated the distinct nature of these models and established them as a valuable and faithful platform for functional, multi-omic and drug screening initiatives. These hold significant potential for uncovering the underlying mechanisms of central features of GBM, such as infiltration and hypoxia. They also provide advanced systems for drug screening and the nomination of new targets for therapeutic efforts. In light of the highly plastic nature of GBM cells and the importance of the interactions within the tumor microenvironment [67], recent and upcoming research using these new models appears to constitute a promising approach. Continued use and further development of these advanced systems can potentially garner important progress and ultimately concrete improvements in the management of patients with glioblastoma.

## Data Availability

Not applicable.

## Abbreviations

CNS: Central nervous system
CO: Cerebral organoid
CTIP2: COUP-TF-interacting protein 2
DAPI: 4′,6-diaminido-2-phenylindole
DCX: Doublecortin
ECM: Extracellular matrix
EGFR: Epidermal growth factor receptor
ETV2: ETS variant transcription factor 2
GBM: Glioblastoma multiforme
GFAP: Glial fibrillary acidic protein
GFP: Green fluorescent protein
GLICO: Glioma cerebral organoid
GSC: Glioblastoma stem cell
hESC: Human embryonic stem cell
hPSC: Human pluripotent stem cell
HRas^G12V^: Mutant HRas where glycine is replaced by valine at position 12
iPSC: Induced pluripotent stem cell
PDX: Patient-derived xenograft
PTPRZ1: Protein Tyrosine Phosphatase Receptor Type Z1
SOX2: SRY-Box Transcription Factor 2
TP53: Tumor protein 53
VEGF: Vascular endothelial growth factor
